# Low-dose PLX5622 treatment prevents neuroinflammatory and neurocognitive sequelae after sepsis

**DOI:** 10.1186/s12974-023-02975-8

**Published:** 2023-12-01

**Authors:** Nils Mein, Nikolai von Stackelberg, Jonathan Wickel, Christian Geis, Ha-Yeun Chung

**Affiliations:** 1 Section of Translational Neuroimmunology, Department of Neurology, Jena University Hospital, Am Klinikum 1, 07747 Jena, Germany; 2 Center for Sepsis Control and Care, Jena University Hospital, 07747 Jena, Germany; 3 German Center for Mental Health, Center for Intervention and Research on Adaptive and Maladaptive Brain Circuits Underlying Mental Health (C-I-R-C), Jena-Magdeburg-Halle, Germany

**Keywords:** Microglia, PLX5622, Sepsis, Encephalopathy, Neurocognitive dysfunction

## Abstract

**Background:**

Sepsis-associated encephalopathy (SAE) is characterized by symptoms of delirium including hallucinations, impaired concentration, agitation, or coma and is associated with poor outcome in the early phase of sepsis. In addition, sepsis survivors often suffer from persisting memory deficits and impaired executive functions. Recent studies provide evidence that microglia are involved in the pathophysiology of SAE.

**Methods:**

Here, we investigated whether pharmacological depletion of microglia using PLX5622 (1200 ppm or 300 ppm) in the acute phase of sepsis is able to prevent long-term neurocognitive decline in a male mouse model of polymicrobial sepsis or lipopolysaccharide-induced sterile neuroinflammation. Therefore, we performed the novel object recognition test at different time points after sepsis to address hippocampus-dependent learning. To further assess synapse engulfment in microglia, colocalization analysis was performed using high-resolution 3D Airyscan imaging of Iba1 and Homer1. We also investigated the effect of PLX5622 on acute astrocyte and chronic microglia proliferation in the hippocampus after sepsis induction using immunofluorescence staining.

**Results:**

High-dose application of the colony stimulating factor 1 receptor (CSF1R) inhibitor PLX5622 (1200 ppm) seven days prior to sepsis induction lead to 70–80% microglia reduction but resulted in fatal outcome of bacterial sepsis or LPS induced inflammation. This is likely caused by severely compromised host immune response upon PLX5622-induced depletion of peripheral monocytes and macrophages. We therefore tested partial microglia depletion using a low-dose of PLX5622 (300 ppm) for seven days prior to sepsis which resulted in an increased survival in comparison to littermates subjected to high-dose CSF1R inhibiton and to a stable microglia reduction of ~ 40%. This partial microglia depletion in the acute stage of sepsis largely prevented the engulfment and microglia-induced stripping of postsynaptic terminals. In addition, PLX5622 low-dose microglia depletion attenuated acute astrogliosis as well as long-term microgliosis and prevented long-term neurocognitive decline after experimental sepsis.

**Conclusions:**

We conclude that partial microglia depletion before the induction of sepsis may be sufficient to attenuate long-term neurocognitive dysfunction. Application of PLX5622 (300 ppm) acts by reducing microglia-induced synaptic attachement/engulfment and preventing chronic microgliosis.

**Supplementary Information:**

The online version contains supplementary material available at 10.1186/s12974-023-02975-8.

## Background

Sepsis is the leading cause of death in intensive care units worldwide with mortality rates up to ~ 50% [1]. It is caused by systemic inflammation leading to dysregulated host response that can ultimately cause multi-organ dysfunction [2, 3]. Sepsis-associated encephalopathy (SAE) is a serious and common complication that manifests with symptoms of acute cerebral dysfunction such as hallucinations, agitation and/or coma [4–6]. The occurrence of SAE in sepsis patients is associated with neuronal injury and poor outcome [7–9]. In addition to symptoms of cerebral dysfunction in the acute stage, SAE also results in long-term neurocognitive sequelae, such as fatigue, impaired concentration and neurocognitive deficits that might resemble mild cognitive impairment [4, 10]. These long-term effects result in reduced quality of life of sepsis survivors and their caregivers and cause a substantial socioeconomic burden [11]. With improvements in intensive care treatment over the last decade and the increase of sepsis survivors, the treatment and prevention of long-term sequelae, such as neurocognitive deficits, has become increasingly relevant in infectious diseases [12, 13].

Microglia constitute the resident, parenchymal macrophages of the central nervous system (CNS) and are involved in the development of symptoms in acute and chronic SAE [14–16]. Using their highly motile processes, they continuously scan their environment and interact with other cells, such as astroglia and neurons [17, 18]. Under physiological conditions microglia have a variety of functions including the control of development, homeostasis and synaptogenesis in the CNS [19, 20]. Several preclinical studies provide evidence that microglia are key players in the pathophysiology of several brain disorders, such as Alzheimer’s disease or traumatic brain injury, thus identifying microglia as a potential therapeutic target [21, 22]. As shown recently, microglia are responsible for the induction and maintenance of neuroinflammation during SAE [23]. Here, microglia present an activated phenotype that triggers long-lasting neuronal damage through increased synaptic engulfment and stripping of synaptic terminals.

The tyrosine kinase transmembrane receptor CSF1R may be a potential target for interfering with microglia during systemic inflammation. The CSF1R is highly expressed on myeloid cells, such as microglia, and its signaling is essential for the survival, proliferation, and differentiation of these cells [24]. Using the pharmacological compound PLX5622, a CSF1R inhibitor and blood–brain barrier penetrating molecule, microglia can be effectively depleted [25–27]. PLX5622 is available in two dosages: 300 mg/kg and 1200 mg/kg. Both dosing strategies cause a decrease of microglia population in the adult murine brain resulting in 40% loss of microglia after low-dose (300 mg/kg) and 80% loss of microglia after high-dose (1200 mg/kg) PLX5622 diet [28]. Moreover, this compound has been shown to improve disease-associated symptoms in several neurological diseases in which microglial activity is critical [21, 26, 29, 30]. Interestingly, PLX3399—a related pharmacological compound—has already been approved by the FDA and is currently used under the name of pexidartinib to treat patients with symptomatic tenosynovial giant cell tumors [31]. Very recently, we have shown that microglia depletion using high-dose PLX5622 in experimental model of polymicrobial sepsis at a subacute and late stage results in improvement of synaptic loss and cognitive dysfunction [23].

In this study, we used several interventional strategies in mouse models of polymicrobial sepsis or lipopolysaccharide- (LPS) induced inflammation to investigate whether pharmacological depletion of microglia in the very acute phase of sepsis has the potential to prevent the cascade of pathogenic neuroinflammation and to preserve neurocognitive function.

## Materials and methods

### Animals

All experiments were performed in accordance with the ARRIVE guidelines 2.0 (Additional file 1: Table S1) [32], with the German legislation on protection of animals and with approval of the local animal welfare committee (Thüringer Landesamt für Lebensmittelsicherheit und Verbraucherschutz, UKJ-18-026). A total of 7 experiments were performed using 193 male C57BL/6J mice, aged 10–13 weeks, from the in-house breeding facility. Animals were randomized to the treatment groups, with equal distribution of age and weight in all four groups. Animals were housed under standardized day-night conditions (12 h/12 h) at room temperature (23 ± 1 °C, 30–60% humidity). Mice were fed with AIN-76A (Research Diets, Inc.) standard chow and water ad libitum.

### Peritoneal contamination and infection (PCI) sepsis model and PLX5622 treatment

Polymicrobial sepsis was induced by the standardized peritoneal contamination and infection model (PCI) and sterile systemic inflammation using lipopolysaccharide (LPS) administration [33, 34]. Human fecal slurry in the PCI model (diluted 1:4 in saline solution; 3.5 µl/g B.W.) or LPS (3 mg/kg B.W.; Sigma-Aldrich, Germany) were injected into the left lower quadrant of the abdomen with a 21-gauge cannula. To increase survival rates and ensure comparability, sham and PCI treated animals received subsequent antibiotic treatment. Therefore, meropenem (20 mg/kg B.W.) was injected s.c. every 12 h for 7 days starting at a clinical severity score (CSS) of 3, indicating severe infection, in both sham and PCI mice [33]. Between day 8–10 meropenem was applied once daily. Thereafter, enrofloxacin (Baytril 2.5%, Bayer AG, Germany) was diluted in saccharose containing drinking water (final concentration of 2 mg/ml) and applied until the end of the experiment to avoid re-occuring infection [23]. Disease severity was continuously assessed using the Clinical Severity Score (CSS; grade 1–4) [33]. Grade 1: No signs of illness, active and curious, quick movements upon exogenous stimuli, normal posture; grade 2: Low-grade illness, less active with occasional interruptions in activity, reduced alertness, but adequate response to exogenous stimuli, posture slightly hunched; grade 3: moderately severe illness, slow and sleepy, movement difficulty, limited and delayed reaction to exogenous stimuli, hunched posture; grade 4: severe illness, mouse lethargic, motionless, no spontaneous movements, no reaction to exogenous stimuli, severely hunched posture. Floating numbers between two grades are possible. For ethical reasons, mice were euthanized if a CSS of grade 4 was reached at two consecutive time points within 3 h and mice were not included in further analysis. To ensure adequate and comparable disease severity between groups, only mice with a 3-day cumulative CSS greater than 9.5 and a 5-day cumulative CSS greater than 10.5 were included in the study for further analysis.

PLX5622 was provided by Plexxikon Inc. and is formulated in AIN-76A standard chow in given dosages of 1200 mg/kg and 300 mg/kg. PLX5622 needs to be administrered for at least 7 days to achieve stable microglia depletion [26, 28]. High-dose PLX5622 (1200 ppm; 1200 mg/kg; Plexxicon Inc. Berkeley, CA, USA) or low-dose PLX5622 (300 ppm; 300 mg/kg; Plexxicon Inc. Berkeley, CA, USA) containing AIN-76A (AIN) standard chow in the microglia depletion groups was started seven days prior to PCI or LPS induction in comparision to control diet AIN. At the time point of PCI induction or LPS administration PLX5622 treatment was ended. Mice were continuously weighed before and after sepsis induction or LPS administration to assess adequate uptake of PLX5622 and to evaluate disease severity during sepsis.

### Immunohistochemistry

At the end of experiments, animals were sacrificed by overdosage of isoflurane (2%) and were transcardially perfused with phosphate buffered saline (PBS). Brain tissue was harvested for further *ex-vivo* experiments at the respective time points. After preparation, the dissected brains were cut in half and one hemisphere was fixated for 24 h in 4% paraformaldehyde (PFA), dehydrated for 24 h in 10% and 24 h in 30% sucrose solution. The PFA-fixed hemispheres were stored at − 80 °C. Coronal sections were prepared with a cutting microtome (16 or 40 µm) (Thermo Scientific Microm HM 450, Thermo Fischer Scientific, Schwerte, DE, USA) and stored at − 20 °C as free-floating sections in antifreeze solution.

For immunohistochemistry, slices were washed with TRIS buffered saline (TBS) and blocked with blocking buffer (3% serum, 2% milk powder and 0.1% Triton X-100 in TBS) for 30 min. Thereafter, slices were incubated overnight at 4 °C with the primary antibody against the ionized calcium-binding adapter molecule 1 (Iba1) (rabbit, 1:500, Cat# 019-19741, RRID:AB_839504, FUJIFILM Wako Shibayagi), the glial fibrillary acidic protein (GFAP) (mouse, 1:500, Cat# MAB360, RRID:AB_11212597, Millipore) or Homer1 (chicken, 1:500, Cat# 160006, RRID:AB_2631222, Synaptic Systems). Following washing steps in TBS, slices were incubated for 2 h in the secondary antibody (AF488, donkey@rabbit, 1:500, Cat# A-21206, RRID:AB_2535792, Molecular Probes; AF488, 1:200, goat@chicken, Cat# A-11039, RRID:AB_142924, Thermo Fisher Scientific; AF647, 1:500, donkey@ms, Cat# 715-605-151, RRID:AB_2340863, Jackson ImmunoResearch Labs; AF647, 1:500, goat@rabbit, Cat# A-21244, RRID:AB_2535812, Thermo Fisher Scientific). Following washing in TBS, slices were transferred to microscope slides with 0.5% gelatin, dried and stained for 5 min in 4′,6-Diamidino-2-phenylindol (DAPI) (Sigma Aldrich, Cat# D9542, CAS-Nr. 28718-90-3, 1 mg/ml) solution and washed in PBS. Cover slips were mounted with Fluoromount G (Southern Biotech, Birmingham, AL, USA). Immunostained brain slices were imaged with a confocal laser scanning microscope (Zeiss LSM 900). For colocalization analysis regarding the synaptic engulfment assay the Airyscan mode of the microscope providing high-resolution images was used.

### Pharmacological microglia depletion and microgliosis

For quantitative microglia analysis, Iba1 positive cells were counted in the CA1 region of the hippocampus. Therefore, murine brain sections (40 μm section thickness) were subsequently stained with commercial antibodies against Iba1 and DAPI. Representative Z-stack images were obtained (20 × objective, NA = 0.8). Z-stacks were scanned at 2048 × 2048 resolution, with 4 pixel-wise plane scan intensity averages for each color channel, and 1 μm step size to generate confocal stacks of ~ 5 μm per image. Finally, maximum intensity projections were generated and Iba1 microglia were manually counted in Fiji ImageJ 2.3.0 [27]. Only Iba1 positive cells with a DAPI-positive nucleus were counted. For the overview images tile scans (20 × objective, NA = 0.8) were generated. Cell count was normalized to the average cell count of the control condition AIN-sham. Experimenters were blinded to the group allocation.

### GFAP-staining and quantitative analysis

For astrocyte analysis, slices were stained for GFAP and DAPI. To quantify hippocampal astrocytes (20 × objective, NA = 0.8) z-stacks were taken in the CA1 region (d3: n = 5 animals/group, n = 3 images/animal; d38: n = 5 animals/group, n = 2 images/animal). A standardized z-stack thickness of 20 µm with a z-interval of 1 µm per plane was used for each color channel. Cells were manually quantified using Fiji. Only GFAP-positive cells with a DAPI-positive nucleus in the center plane at 3 µm of the image stack were counted. Cell count was normalized to the average cell count of the control condition AIN-sham. Experimenters were blinded to the group allocation.

### Quantitative analysis of engulfed synapses

For the colocalization analysis of synaptic engulfment, slices were stained for Iba1, Homer1 and DAPI. Representative z-stack images (d3: n = 4–5/group, n = 2–4 microglia/animal; d38: n = 5/group, n = 2microglia/animal) were acquired in the CA1 region of the hippocampus (63 × oil objective, NA = 1.4, Airyscan). Z-stacks were acquired with a 1490 × 1490 image size and with 4 pixel-wise plane scan intensity averages for each color channel and 16.66 µm z-step size. After image acquisition, "surfaces" were created in Imaris (version 9.1.2.; Oxford instruments) for Iba1 and Homer1. Using the "colocalization" function and an implemented MatLab code in Imaris, a fluorescence colocalization of Iba1 and Homer1 was performed. In the following, a mean threshold filter of 0.1 µm^3^ was applied to the colocalized volumes, based on the mean volumes of the colocalized volumes of both groups.

### Open field test

Exploratory activity and anxiety-like behavior following sepsis induction were measured by open field test. The open field test was performed on day 9 and 29 after PCI induction one day prior to each novel object recognition (NOR) test. The experiment was conducted in a black open-field box (40 cm), illuminated by 15 lx LEDs, by an experimenter blinded to the group allocation of the two intervention strategies—PCI injection and PLX5622 treatment. Mice were placed in the open field box for 10 min (5 min for day 29) and were able to discover the box freely. The mean velocity and total distance were measured automatically on each day using the tracking software EthoVision (Ethovision XT^®^ 6.1 software (Noldus Wageningen Netherlands) [35]. Between each trial, the box was carefully cleaned with 70% ethanol to ensure equal experimental conditions.

### Novel object recognition test

To test learning and memory after sepsis, novel object recognition (NOR) test was performed as described previously [23]. NOR was performed at day 10 and 30 after PCI induction. The experiment was conducted in a black open-field box (40 cm), illuminated by 15 lx LEDs, by an experimenter blinded to the group allocation of the two intervention strategies—PCI injection and PLX5622 treatment. Objects were distinguishable and different for each testing day: an iron bracket and an objective container (both day 10), a small plastic cylinder, and an iron eyebolt (both day 30). The objects were previously tested and no preferences for an individual object could be found. Additionally, each object was randomized to be used as familiar or novel object. Between each trial, the box was carefully cleaned with 70% ethanol to ensure equal experimental conditions.

During the familiarization phase, mice were allowed to explore two equal objects for max. 10 min. The familiarization phase was terminated by the investigator after mice had explored the two objects for cumulative 20 s or 10 min if a cumulative exploration time of 20 s was not reached. In the testing phase, 6 h after the training phase, one object was replaced by the complementary novel object, and mice were allowed to explore the familiar and new object. The position of the object, left vs. right, was also randomized between animals during the test phase. Exploration time was measured and the session was completed after 20 s cumulative exploration behavior. Mice, which did not explore the objects for 20 s within 10 min in the familiarization or testing phase were excluded from the analysis. Every touching of the object and direct facing an object ≤ 2 cm apart from the object was considered as exploration behavior. Climbing onto the object was not regarded as exploration behavior.

### Statistics

**Fig. 1:**
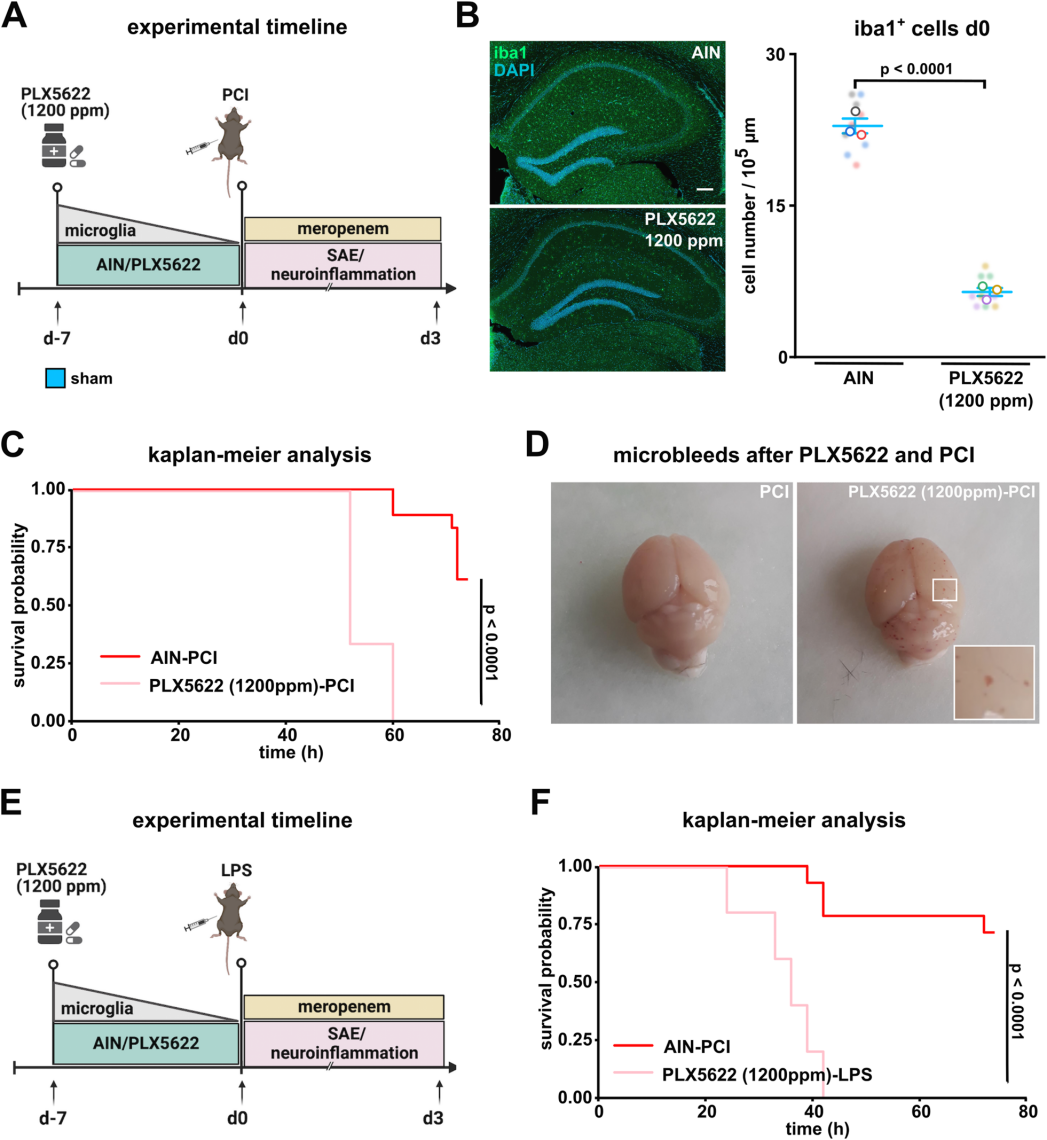
High-dose PLX5622 pretreatment causes poor outcome in LPS and PCI induced systemic inflammation. **A** Timeline of experiment: mice were randomized to AIN or PLX5622 (1200 ppm) treatment seven days prior to PCI induction. **B** Quantitative analysis of Iba1-positive microglia in the CA1 region of the hippocampus at day 0 following AIN or PLX5622 treatment (n = 3 animals/group; n = 3 images/animal, Student’s t-test). Representative confocal overview images of microglia in the CA1 region after pretreatment with either AIN or high-dose PLX5622 (1200 ppm) on day 0. Scale bar 200 µm. **C** Kaplan–Meier analysis of PCI induced sepsis following AIN or high-dose PLX5622 treatment for seven days (AIN: PCI: n = 21 animals/group, PLX5622: PCI: n = 6 animals/group, log-rank test). **D** Representative photograph of cerebral microbleeds in mice pretreated with high-dose PLX5622 for seven days followed by PCI induction. **E** Timeline of experiment: mice were randomized to AIN or high-dose PLX5622 (1200 ppm) treatment seven days prior to induction of systemic inflammation by LPS. **F** Kaplan–Meier analysis of LPS induced systemic inflammation following AIN and high-dose PLX5622 pretreatment for 7 consecutive days (AIN: LPS: n = 14 animals/group and PLX5622: LPS: n = 6 animals/group). Data are presented as mean ± SEM. Individual values are presented as small dots and each circle represents an average of one mouse; individual values and averages are color coded

**Fig. 2:**
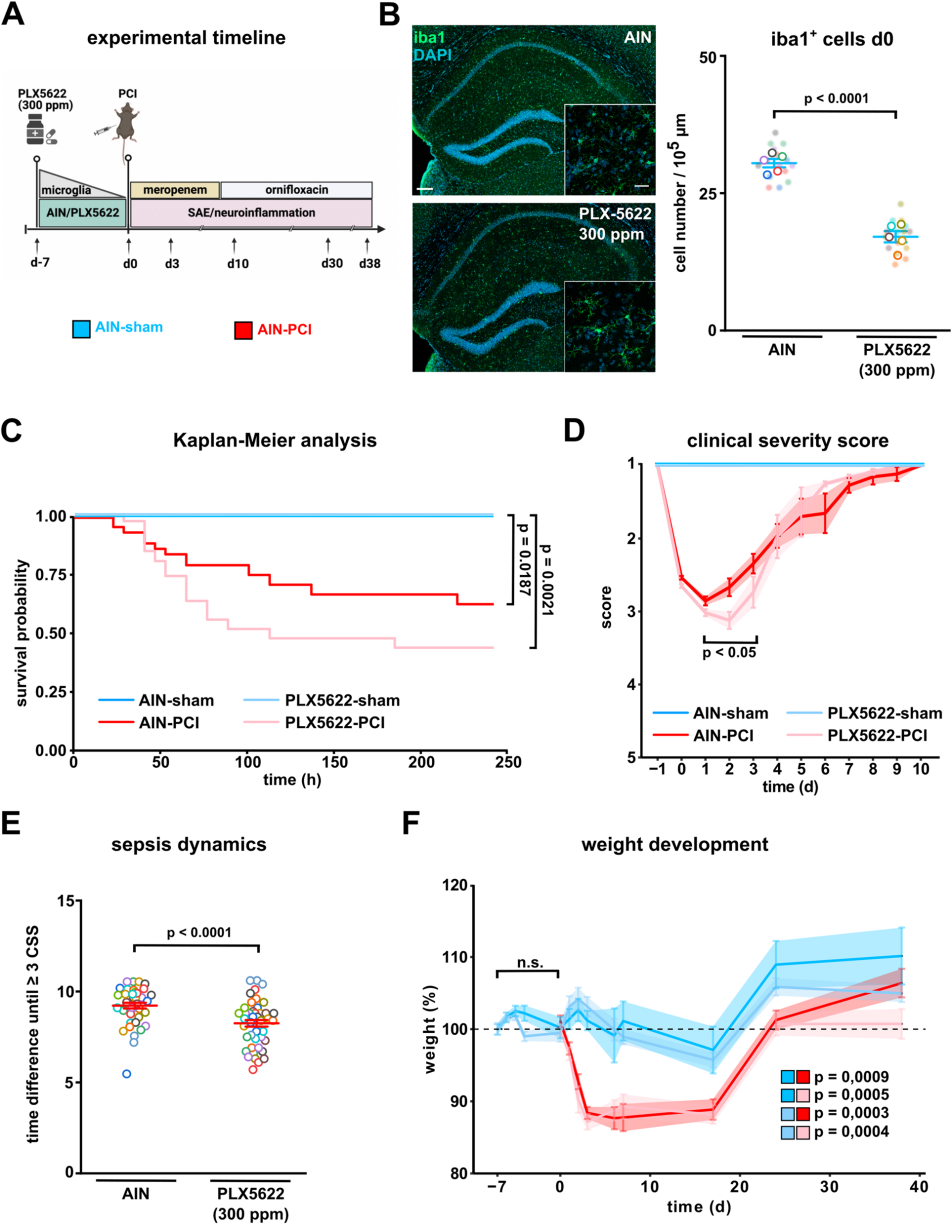
Partial microlgia depletion by low-dose PLX5622 only slightly affects the course of sepsis. **A** Timeline of experiment: mice were randomized to AIN or low-dose PLX5622 (300 ppm) treatment seven days prior to PCI. After PCI (day 0) all mice received standard AIN chow. **B** Quantitative analysis of Iba1-positive microglia in the CA1 region of the hippocampus following AIN or PLX5622 treatment at day 0 (n = 3 animals/group; n = 15 images/animal, Student’s t-test). Representative confocal overview and zoom-in images of Iba1-positive microglia in the CA1 region after pretreatment with either AIN or PLX5622 (300 ppm). Scale bar 20 µm and 200 µm. **C** Kaplan–Meier analysis of PCI induced systemic inflammation following AIN and low-dose PLX5622 between day 0 until day 38 (AIN-sham: n = 20, AIN-PCI: n = 42; PLX5622-sham: n = 20, PLX5622-PCI: n = 45, log-rank test: p < 0.0001). **D** Clinical severity score (AIN-sham: n = 20, AIN-PCI: n = 42; PLX5622-sham: n = 20, PLX5622-PCI: n = 45; the adapted two-tailed permutation test of Cohen (significance level of 5%) thereby accounting for the multiple-comparison problem has been performed. **E** Time dynamics in the development of clinical severity score until CSS ≥ 3 (AIN-PCI: n = 42, PLX5622-PCI: n = 45, Mann–Whitney-U-test). **F** Weight development during pretreatment of AIN or low-dose PLX5622 between day -7 until day 0 and during polymicrobial sepsis between day 0 and day 38 (AIN: sham: n = 20, PCI: n = 42; PLX5622: sham: n = 20, PCI: n = 45; day -7 until day 0: Curve-permutation test (two-tailed), p = 0.3780; day 0 until day 38: Curve-permutation test (two-tailed) with Benjamini–Hochberg correction. Data are presented as mean ± SEM. Individual values are presented as small dots and each circle represents an average of one mouse; individual values and averages are color coded

Researchers performing the analyses were blinded to the experimental treatment of the animals. Based on our previous results with the PCI mouse model [23], we performed a statistical power analysis to predict the minimum sample size per treatment group which was found to be close to 6 (p = 0.05 and 80% power). All graphs are represented as super-plots showing means ± standard error of the mean (SEM). Individual values are presented as small dots and each circle represents an average of one mouse. The latter was used for statistical analysis. Analysis was performed using R (1.4.1717) und R Studio (2021.09.0), OriginPro (v. 2020b), SigmaPlot (v. 14.5), and Matlab 2020a. If data were normally distributed (Shapiro–Wilk test), two-tailed unpaired Student’s t-test (2 groups) or Two-way ANOVA (4 groups, post hoc: Tukey test) were used. If data were not normally distributed, Mann–Whitney-U-Test (2 groups) were used. Data were also tested for homogeneity of variance (Levene test) and outliers (Grubb’s test). For Kaplan–Meier analysis log-rank test (post-hoc: Benjamini–Hochberg correction) was performed. Permutation tests were performed based on ref. [36] as follows: to compare the differences in the CSS, we adapted the two-tailed permutation test of Cohen (significance level of 5%), thereby accounting for multiple comparison problem. For the weight development between d-7-d0 and between d0-d38, we performed the two-tailed curve-permutation test. For days d1-d38, we subjected p-values to Benjamini–Hochberg procedure in order to account for multiple comparison problem. For each permutation test, 1 million times shuffling was used. Figures 1A, E, 2A, 6A, D were created with BioRender.com.

## Results

### High-dose PLX5622 before PCI and LPS results in poor outcome after polymicrobial sepsis and LPS induced neuroinflammation

In a first experimental series, we tested if a seven day pretreatment of high-dose PLX5622 is sufficient to reduce brain microglia (Fig. 1A). Here, we found an overall reduction of microglia in the CA1 region of the hippocampus of  ~ 70% (Fig. 1B). We applied PLX6522 or AIN control diet seven days before PCI induction or LPS injection in the following experiments. As expected, when compared to previous studies using PCI in C57BL/6J mice, PCI-induced polymicrobial sepsis resulted in an overall survival of ~ 60% in AIN pretreated controls [23, 37]. However, all animals pretreated with high-dose PLX5622 died in the acute phase of experimental sepsis within the first three days (Fig. 1C). Reflecting both disease severity and the above mentioned fatal outcome, we noticed that high-dose PLX5622 pretreated animals developed an increased number of intracerebral hemorrhages following the induction of polymicrobial sepsis (Fig. 1D). PLX5622 depletes also peripheral immune cells, in particular monocytes and macrophages expressing the CSF1R [38]. Therefore, we next investigated whether these results can be attributed to a limited response (e.g. phagocytosis) to invading living bacteria or if the increased mortality is caused by secondary, immune-mediated effects in response to inflammatory stimuli. To this end, we induced a sterile systemic inflammation without applying reproducing bacteria by using LPS (Fig. 1E). We observed a survival rate of  ~ 70% in AIN-treated controls (Fig. 1F). Interestingly, similar as observed with high-dose PLX5622 pretreated PCI animals, we observed death in all LPS injected animals which were pretreated with high-dose PLX5622 within the first three days after LPS injection (Fig. 1F). This suggests that the critically affected host response depends not only on dysfunctional pathogen defense, but involves also compromised secondary immune effects upon inflammation.

### Low-dose PLX5622 before PCI results in higher acute sepsis severity and trends towards higher mortality after experimental sepsis

Based on our finding of an overall 100% mortality rate in mice treated with high-dose PLX5622 before sepsis, we next performed a pretreatment strategy with low-dose PLX5622 (300 ppm) seven days prior to PCI induction (Fig. 2A). Again, we investigated the degree of microglia depletion following a seven day application of low-dose PLX5622. Here, we observed an ~ 40% reduction in Iba1 positive microglia in the CA1 region of the hippocampus on the day of sepsis induction (Fig. 2B). In the following experiments, we performed PCI or sham application in low-dose PLX5622 treated animals and AIN controls (Fig. 2A). AIN-treated controls showed an overall survival probability of ~ 60% as compared to ~ 46% in low-dose PLX5622-treated animals, which was not significantly different (Fig. 2C). We noticed that low-dose PLX5622 treatment resulted in a slightly higher sepsis severity measured by the CSS (Fig. 2D). In support of these findings, low-dose PLX5622-treated animals reached a CSS ≥ 3, indicating extended sepsis severity, earlier after sepsis induction as compared to littermates treated with AIN control chow (Fig. 2E). In line with these results both AIN-treated PCI and PLX-treated PCI animals showed a weight reduction during the course of sepsis (Fig. 2F). Mice subjected to PLX5622 showed a similar weight development before sepsis induction indicating adequate uptake of PLX5622 (Fig. 2F).

Overall, our data demonstrates a moderate but robust reduction of microglia in a model of low-dose PLX5622 pretreatment. We further observed a higher sepsis severity in PLX5622 pretreated animals with a trend towards higher mortality. However, we could not detect a significant difference in the overall survival rate following sepsis induction. We therefore decided to use low-dose PLX5622 to investigate the effect of partial pharmacological microglia depletion at the onset of systemic inflammation on neurocognitive outcome.

### Partial pharmacological microglia depletion in the acute stage attenuates long-term microgliosis and acute astrogliosis after sepsis

**Fig. 3:**
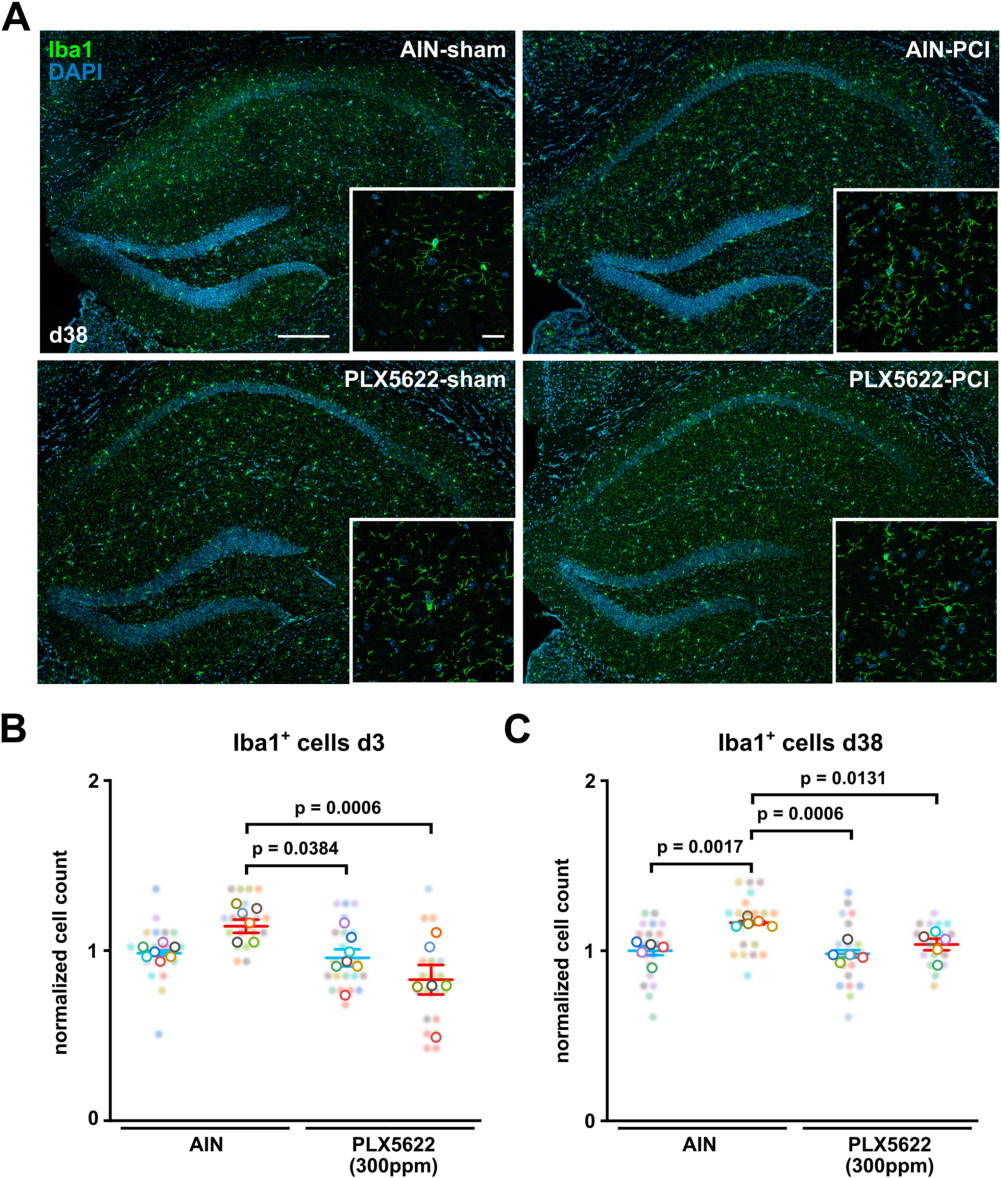
Partial microglia depletion by low-dose PLX5622 prevents acute and long-term microgliosis after polymicobial sepsis. **A** Representative confocal images of microglia in the CA1 region of the hippocampus at day 38. Scale bar 200 µm, zoom-in 20 µm. **B** Quantitative analysis of Iba1-positive microglia in the CA1 region of the hippocampus following AIN or low-dose PLX5622 pretreatment and systemic inflammation by PCI at day 3 (AIN-sham: n = 7, AIN-PCI: n = 6; PLX5622-sham: n = 7, PLX5622-PCI: n = 6; n = 3 images/animal, Two-way ANOVA with post hoc Tukey test). **C** Quantitative analysis of Iba1-positive microglia in the CA1 region of the hippocampus at day 38 following AIN or low-dose PLX5622 pretreatment and systemic inflammation by PCI (n = 5 animals/group, n = 4 images/animal, Two-way ANOVA with post hoc Tukey test). Data are presented as mean ± SEM. Individual values are presented as small dots and each circle represents an average of one mouse; individual values and averages are color coded.

Next, we investigated the effect of partial pharmacological microglia depletion in the CA1 region of the hippocampus at the onset of sepsis induction using low-dose PLX5622. Interestingly, on day three after sepsis induction we still found a reduction of Iba1 positive microglia in the CA1 region of the hippocampus of PLX5622 as compared to AIN treated PCI mice (Fig. 3A, B). Against this, in sham animals pretreated with a low-dose of PLX5622 we already found a normalization of microglia numbers three days after drug withdrawal (from day 0 to day 3) to levels of sham AIN-treated mice. Furthermore, we noticed a long-term microgliosis at day 38 after sepsis induction in AIN treated PCI mice (Fig. 3A, C). Importantly, this long-term microgliosis was prevented in PCI mice, which received low-dose of PLX5622 at the acute stage of sepsis (Fig. 3A, C).

**Fig. 4:**
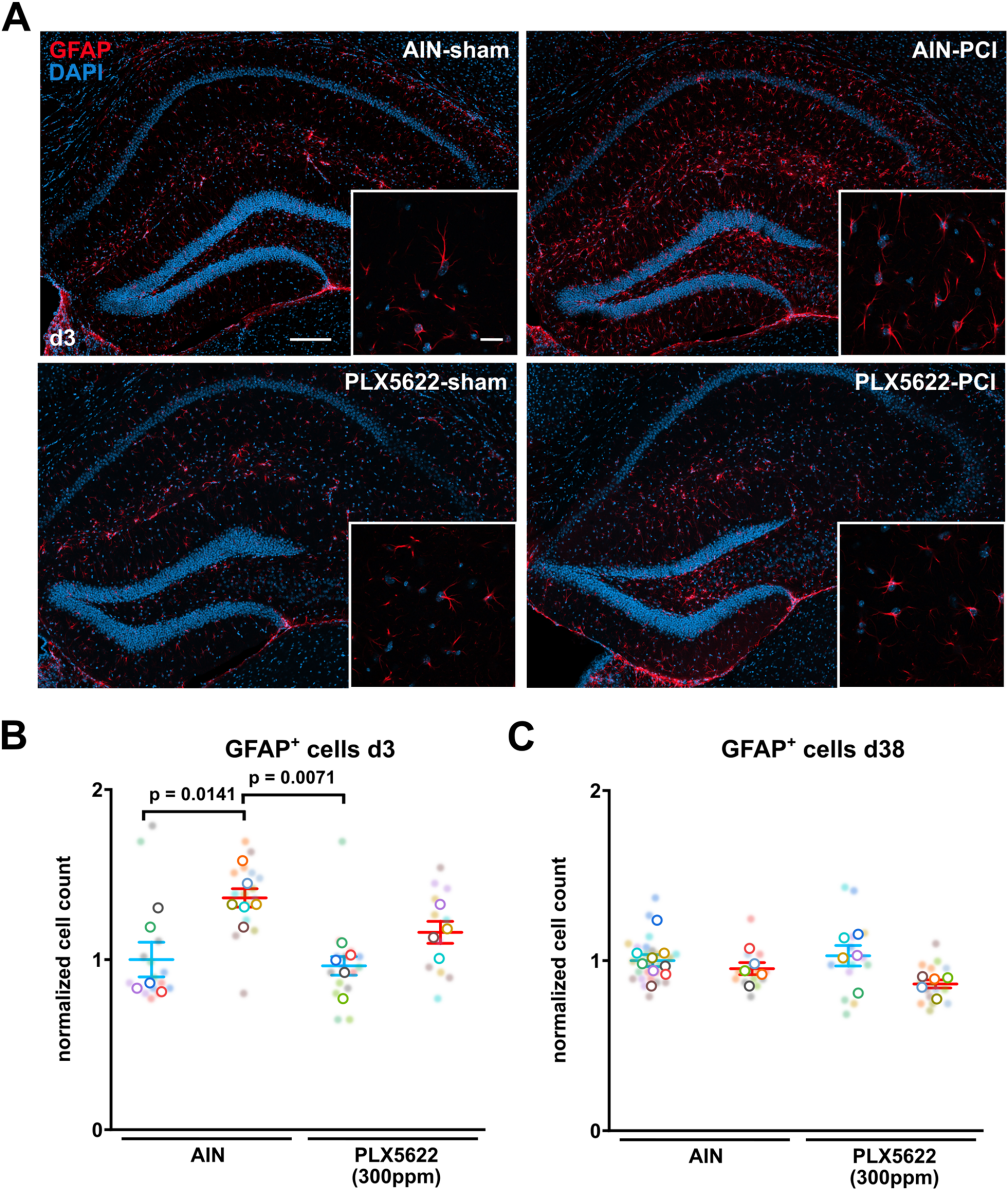
Partial microglia depletion by low-dose PLX5622 prevents acute astrogliosis after polymicobial sepsis. **A** Representative confocal images of astrocytes in the CA1 region of the hippocampus at day 3. Scale bar 200 µm, zoom-in 20 µm. **B** Quantitative analysis of GFAP-positive astrocytes in the CA1 region of the hippocampus following AIN or low-dose PLX5622 pretreatment and systemic inflammation by PCI at day 3 (n = 5 animals/group, n = 3 images/animal, Two-way ANOVA with post hoc Tukey test). **C** Quantitative analysis of GFAP-positive astrocytes in the CA1 region of the hippocampus at day 38 following AIN or low-dose PLX5622 pretreatment and systemic inflammation by (n = 5 animals/group, n = 2 images/animal, Two-way ANOVA with post hoc Tukey test). Data are presented as mean ± SEM. Individual values are presented as small dots and each circle represents an average of one mouse; individual values and averages are color coded

In addition to microglia activation, the glia response upon brain damage often involves parallel astrocyte activation and proliferation [39]. We observed an early reactive astrogliosis on day three after sepsis induction, which was attenuated by pretreatment with PLX5622 (Fig. 4A–C). In the chronic phase at day 38, the number of astrocytes normalized but was still lower in PLX5622 treated PCI mice (Fig. 4C).

Overall, we obsevered that low-dose PLX5622 pretreatment and partial microglia depletion at the acute stage of sepsis prevented chronic microgliosis and acute astrogliosis.

### Pretreatment with low-dose PLX5622 abrogates microglia mediated synaptic microglia attachement and pruning in the acute phase of sepsis

**Fig. 5:**
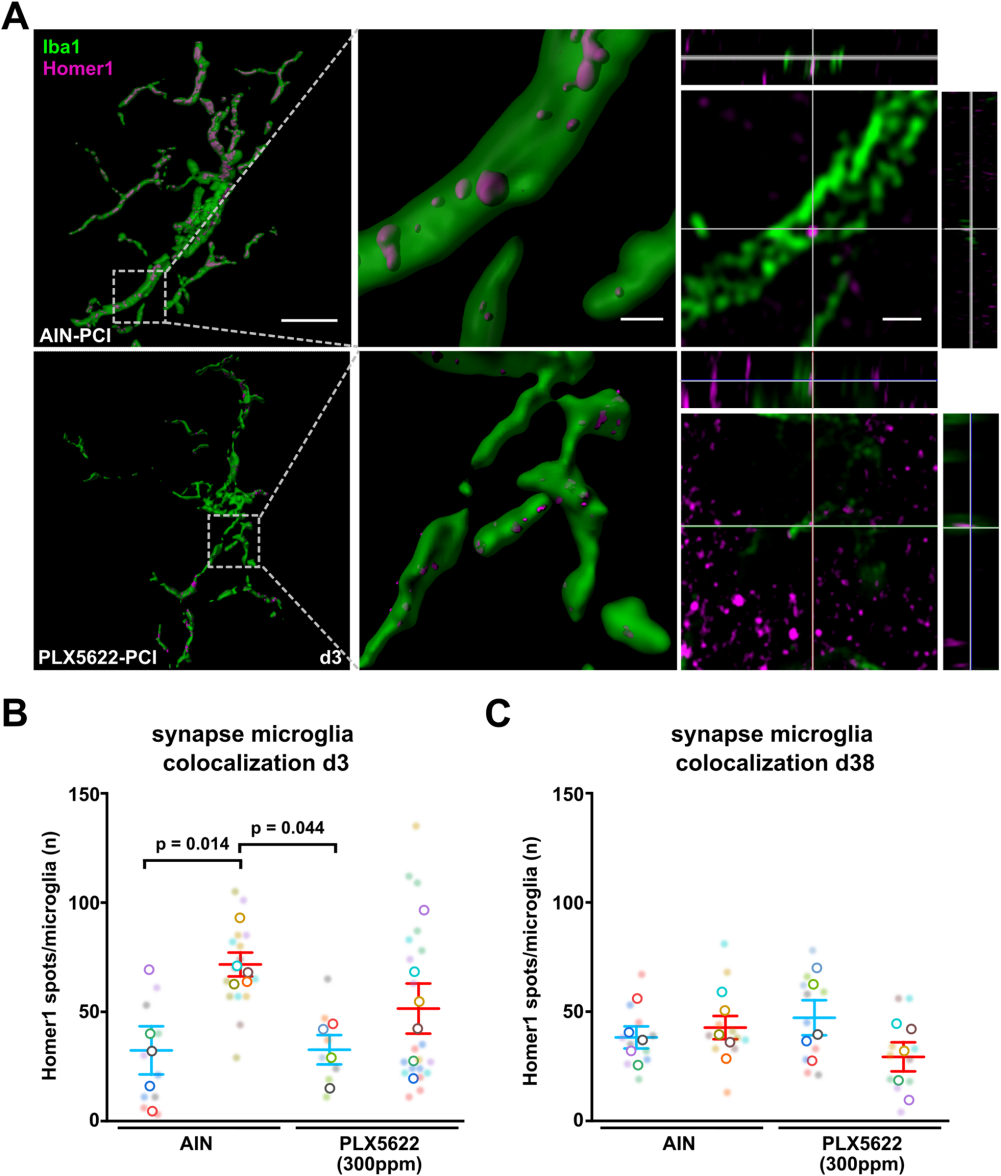
Partial microglia depletion by low-dose PLX5622 prevents sepsis-induced synaptic elimination. **A** Representative 3D reconstructions and Airyscan processed confocal images of Homer1 postsynaptic terminals colocalized with Iba1-positive microglia. Scale bar 10 µm, 1 µm and 2 µm. **B** Quantitative analysis of colocalized Homer1 puncta and Iba1-positive microglia indicating synaptic engulfment or attachment of postsynaptic terminals by microglia in early systemic inflammation on day 3 (n = 4–6 animals/group, n = 3–4 microglia/animal, Two-way ANOVA with post hoc Tukey test). **C** Quantitative analysis of colocalized Homer1 puncta and Iba1-positive microglia indicating synaptic engulfment or attachment of postsynaptic terminals by microglia in chronic systemic inflammation on day 38 (n = 5 animals/group, n = 2 microglia/animal, Two-way ANOVA with post hoc Tukey test). Data are presented as mean ± SEM. Individual values are presented as small dots and each circle represents an average of one mouse; individual values and averages are color coded

Recently, it has been shown that neurocognitive dysfunction after sepsis is caused by excessive microglia-driven synaptic pruning during SAE [23]. Corroborating these results, we found increased synapse attachement and engulfment of postsynaptic Homer1 positive terminals by activated microglia during the early phase of SAE on day three after PCI induction in AIN treated mice (Fig. 5A, B). Remarkably, PCI animals pretreated with PLX5622 showed no significant increase in synaptic attachement and engulfment as compared to controls (Fig. 5A, B). Even though we obserserved a higher number of microglia after sepsis at late stage after PCI (Fig. 3C), we found no differences in the number of attached or engulfed synapses per microglia in the chronic phase of SAE at day 38 following sepsis induction (Fig. 5C).

Our data suggest that the pharmacological depletion of microglia at the onset of sepsis results in a reduced acute and chronic neuroinflammatory response, which attenuates distinct immuno-neuronal effects as evidenced by reduced microglia-dependent synaptic pruning.

### Partial microglia depletion in the acute phase of sepsis prevents from neurocognitive deficits

**Fig. 6:**
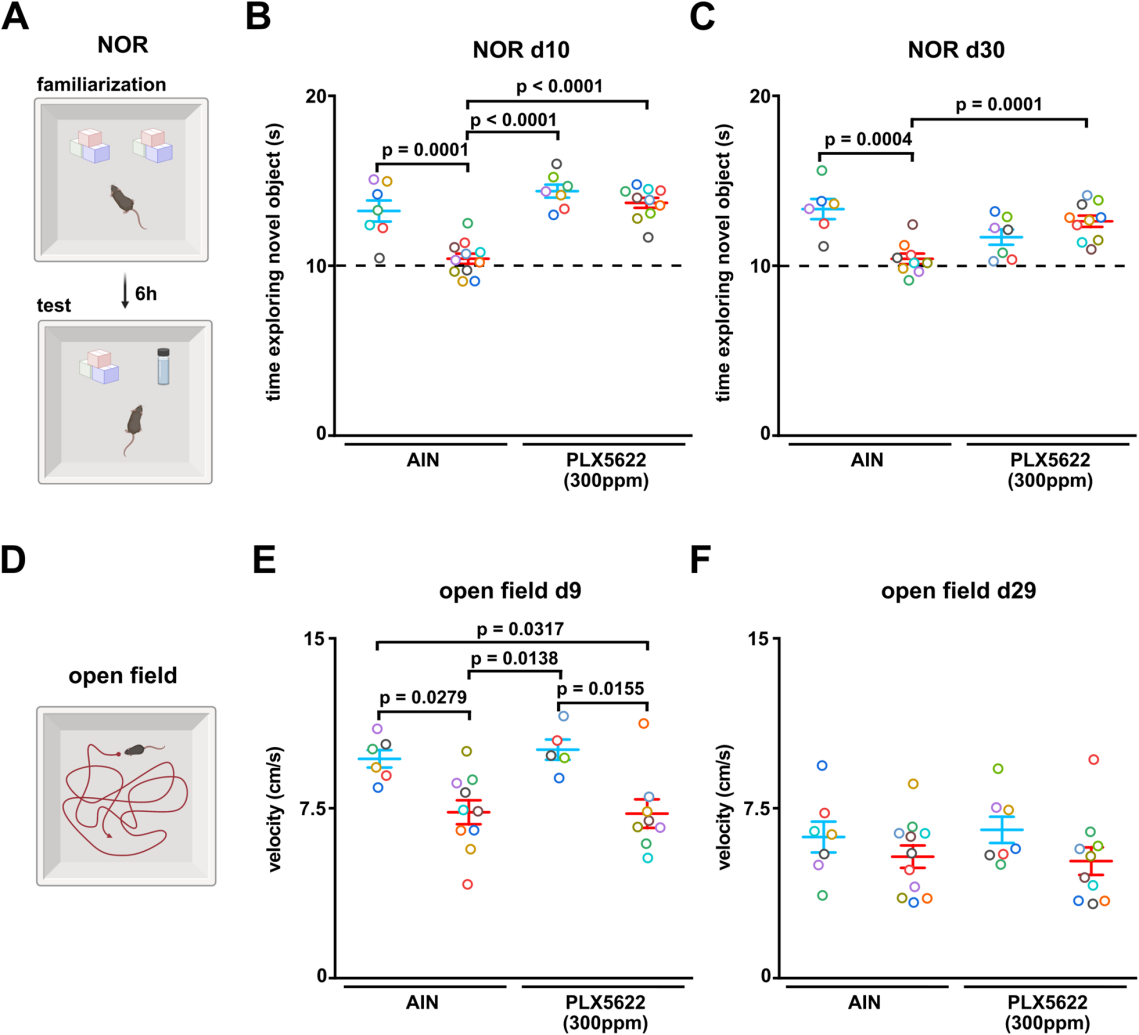
Partial microglia depletion by low-dose PLX5622 rescues from long-term neurocognitive deficits.** A** Schematic NOR test procedure: Mice were familiarized with two identical objects during familiarization. After 6 h intersession interval one object was replaced by the novel object and the exploration time of the novel object was investigated. **B, C** NOR test was performed at day 10 (AIN-sham: n = 7, AIN-PCI: n = 11; PLX5622-sham: n = 7, PLX5622-PCI: n = 10, Two-way ANOVA with post hoc Tukey test) and day 30 (AIN-sham: n = 6, AIN-PCI: n = 9; PLX5622-sham: n = 7, PLX5622-PCI: n = 10, Two-way ANOVA with post hoc Tukey test). **D** Schematic OF test procedure: Mice explored the OF freely and mean velocity has been measeured. **E, F** Open field was performed to analyze the locomotor activity at day 9 (AIN-sham: n = 6, AIN-PCI: n = 10; PLX5622-sham: n = 5, PLX5622-PCI: n = 8, Two-way ANOVA with post hoc Tukey test) and day 29 (AIN-sham: n = 7, AIN-PCI: n = 11; PLX5622-sham: n = 7, PLX5622-PCI: n = 10, Two-way ANOVA with post hoc Tukey test). Data are presented as mean ± SEM. Each circle represents one mouse

In a next step, we investigated the effect of early partial microglia depletion on sepsis-associated long-lasting neurocognitive deficits. We performed novel object recognition tests at two time points to analyze learning and memory abilities (Fig. 6A). PCI animals treated with AIN control chow spent less time exploring the novel object during the test phase at day 10 and 30. In contrast, we observed that littermates, which were subjected to PCI and pretreated with PLX5622, showed better learning and memory function as compared to AIN-treated PCI mice at both time points (Fig. 6B, C and Additional file 1: Fig. S1A, B). In order to investigate whether this behavior was due to reduced locomotor activity, we performed a modified open field test measuring the average velocity and total distance of animals after sepsis induction (Fig. 6D). As expected, PCI animals showed reduced locomotor activity on day 9 with normalized activity on day 29. However, we found no differences between AIN and PLX5622 pretreated PCI animals at either time point (Fig. 6E, F; Additional file 1: Fig. S1C, D).

We conclude that pharmacological microglia depletion by PLX5622 prevented neurocognitive deficits at the onset of the systemic inflammation that could not be explained due to reduced locomotor activity.

## Discussion

In this study, we demonstrate that pharmacological microglia depletion using low-dose PLX5622 improved the neurocognitive outcome after induction of PCI, whereas high-dose PLX5622 resulted in the death of all animals. Low-dose pharmacological microglia depletion attenuated sepsis-induced synaptic pruning as well as reactive microgliosis and astrogliosis.

Recently, microglia have been shown to mediate neurocognitive deficits by eliminating C1q tagged synapses [23]. In this study, a treatment strategy was establisthed using PLX5622 (1200 ppm) three days after sepsis. However, no information regarding a possible benefit of pharmacological microglia depletion in the very beginning of the infection was obtained [23]. We here demonstrate that early microglia depletion during SAE attenuated microglia driven synaptic pruning in the acute phase of SAE and is sufficient to reduce neurocognitive dysfunction. Interestingly, we also observed a chronic microgliosis as a long-term consequence of sepsis, which was not occurring in animals treated with the CSF1R inhibitor. Given the higher number of activated microglia in the chronic phase, we speculate that an increased number of synapses are chronically stripped by microglia, ultimately contibuting to neurocognitive deficits after sepsis. The therapeutic potential may be limited by a moderate worsening of clinical symptoms and survival that we observed using low-dose PLX5622 (300 ppm). In order to avoid any bias in further experiments caused by different survival rates, we only included mice with similar clinical scores (CSS 3 days > 9.5 or 5 days > 10.5) in the treated and untreated PCI groups to ensure adequate and comparable disease severity. A limitation of this study is that low-dose PLX5622 treatment was started before the induction of sepsis to achieve microglia depletion already in the very acute phase of sepsis. For better preclinical evaluation, future studies should investigate different treatment strategies, starting quickly effective drug application during sepsis and adjusting the duration of drug treatment. The CSF1R is not specifically expressed on microglia, but on all myeloid cells including monocytes and macrophages. Therefore it is possible that interfering with the peripheral immune cells may have beneficial effects on the neurocognitive outcome of PLX5622 treated mice independent of microglia depletion [38]. Based on our previous work with detailed subcellular localization analysis showing synaptic pruning and lysosomal degradation of synaptic terminals during PCI, we assume that iba1 colocalized homer1 positive synapses reflect microglia engulfed synapses [23]. However, we cannot fully exclude here that homer1 positive synapses are attached to microglia processes in order of being phagocytosed or are localized in phagosomal or endosomal compartments prior to lysosomal degradation [40].

Pretreatment of mice with a high-dose of PLX5622 (1200 ppm) resulted in a poor outcome, with all animals dying after induction of polymicrobial sepsis. In addition, high-dose PLX5622-treated animals showed multiple haemorrhages in the brain parenchyma, which have also been described in MRI and human post-mortem studies [6, 41]. Haemorrhages are most likely to occur in patients with severe sepsis and/or septic shock, thus corroborating the lethal degree of sepsis in high-dose PLX5622-treated PCI mice. These haemorrhages are likely to be caused by coagulopathy, thrombocytopenia and/or disseminated intravascular coagulation [41, 42]. High mortality after PLX5622 treatment was also observed when we applied LPS to model of non-infectious systemic inflammation. This rather suggests a dysfunctional immunomodulatory host response than a lack of pathogen elimination leading to poor outcome in both, the PCI and LPS model. This observation is consistent with studies showing that pharmacological microglia depletion during systemic viral infection is lethal and several studies have shown that microglia depletion worsens the outcome in viral encephalitis [43, 44]. However, the effect of PLX5622 pretreatment in a model of systemic inflammation without CNS pathogen infiltration has not been described so far. Recent findings provide evidence that PLX5622 severely affects peripheral immune cells, e.g. macrophages and monocytes [38, 45]. Thus, we speculate that the innate immune system is severely compromised and leads to a worse clinical outcome in these animals [38, 44, 46]. However, it should also be noted that CSF1R is expressed on macrophages that patrol the borders of the CNS. It is known that PLX5622 treatment leads to a depletion of border-associated macrophages, which are essential for maintaining the integrity of the CNS and the blood–brain barrier in neurological diseases [47]. In this line, disrupted CNS interfaces and blood–brain barrier may underlie the observed microbleeds seen in PCI mice with high-dose PLX5622 treatment.

We conclude that pharmacological microglia depletion with low-dose PLX5622 in the early infection is sufficient to attenuate clinically relevant brain pathology after sepsis, such as acute astrogliosis, long-term microgliosis and neurocognitive deficits. However, its therapeutic use is limited due to its non-specific effects on peripheral immune cells. Further pre-clinical studies are needed to evaluate the possibility of targeting microglia in sepsis to prevent long-term brain dysfunction and cognitive decline.

### Supplementary Information

**Additional file 1: Fig. S1. **Microglia depletion attenuates long-term neurocognitive deficits independent of locomotor behaviour.** Table S1: **ARRIVE Essential 10 checklist.

## Data Availability

The datasets used and/or analysed during the current study are available from the corresponding author on reasonable request.

## Abbreviations

CSF1R: Colony stimulating factor 1 receptor
SAE: Sepsis-associated encephalopathy
CNS: Central nervous system
PCI: Peritoneal contamination and infection
LPS: Lipopolysaccharide
CSS: Clinical severity score
TBS: TRIS buffered saline
PBS: Phosphate buffered saline
PFA: Paraformaldehyde
Iba1: Ionized calcium-binding adapter molecule 1
GFAP: Glial fibrillary acidic protein
DAPI: 4′,6-Diamidino-2-phenylindol
OF: Open field
NOR: Novel object recognition
