# Supplementary material for: Low-dose PLX5622 treatment prevents neuroinflammatory and neurocognitive sequelae after sepsis
Source: J Neuroinflammation. 2023 Dec 1;20:289. doi: 10.1186/s12974-023-02975-8 (PMC10691003; doi:10.1186/s12974-023-02975-8)
Supplement: Supplementary file 1 — Additional file 1: Fig. S1. Microglia depletion attenuates long-term neurocognitive deficits independent of locomotor behaviour. Table S1: ARRIVE Essential 10 checklist. [file 12974_2023_2975_MOESM1_ESM.docx]

**Additional file 1**

**Additional figures/tables and legends**

**Fig. S1**


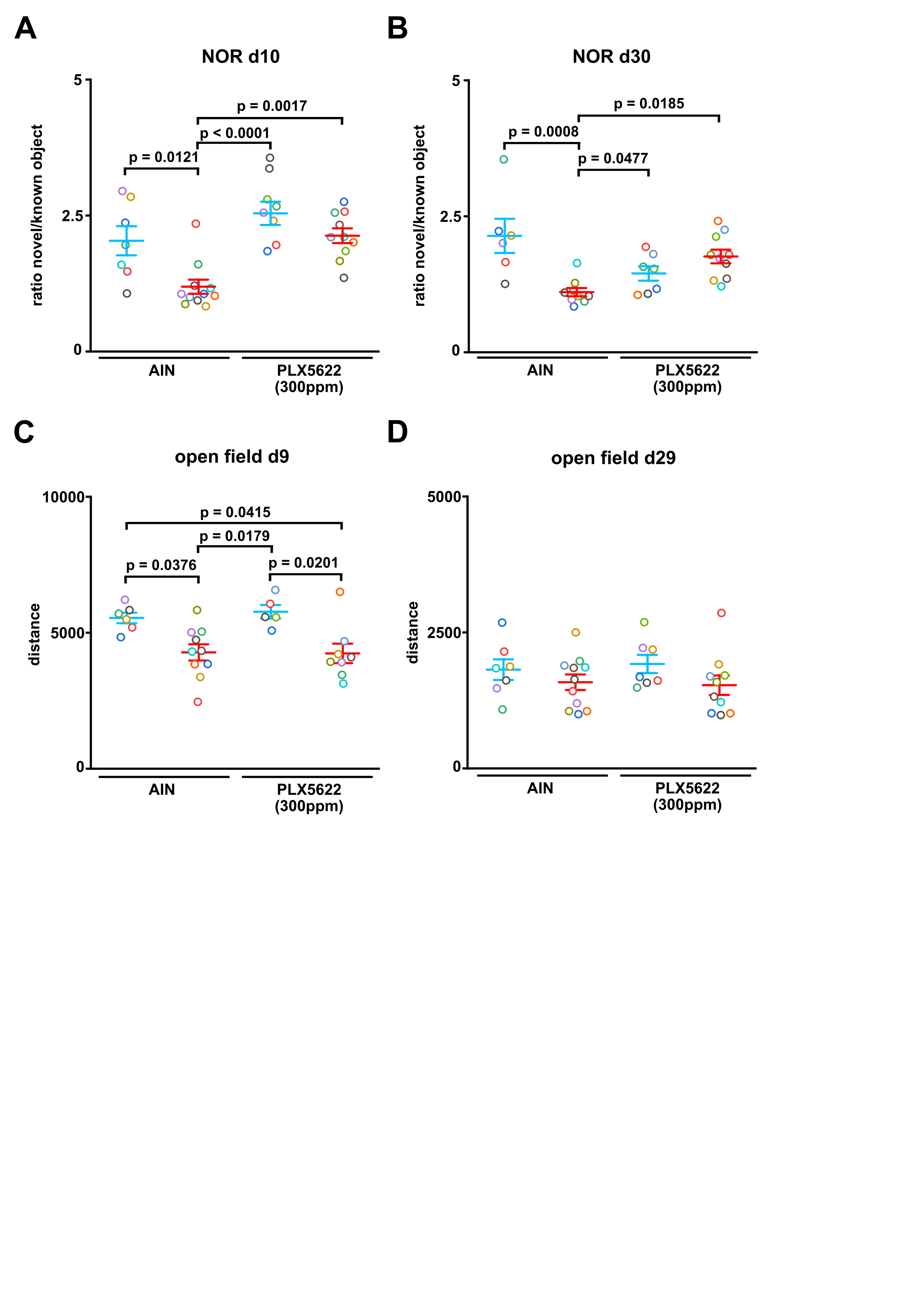


**Fig. S1: Microglia depletion attenuates long-term neurocognitive deficits independent of locomotor behaviour**

**(A-B)** NOR ratio novel/known object at day 10 (AIN-sham: n = 7, AIN-PCI: n = 11; PLX5622-sham: n = 7, PLX5622-PCI: n = 10, Two-way ANOVA with post hoc Tukey test) and day 30 (AIN-sham: n = 6, AIN-PCI: n = 9; PLX5622-sham: n= 7, PLX5622-PCI: n = 10, Two-way ANOVA with post hoc Tukey test). (**C-D)** Total distance in open field test at day 9 (AIN-sham: n = 6, AIN-PCI: n = 10; PLX5622-sham: n = 5, PLX5622-PCI: n = 8, Two-way ANOVA with post hoc Tukey test) and day 29 (AIN-sham: n =7, AIN-PCI: n = 11; PLX5622-sham: n = 7, PLX5622-PCI: n = 10, Two-way ANOVA with post hoc Tukey test).

Data are presented as mean ± SEM. Each circle represents one mouse.

**Table S1**

| **Item** | **Recommendation** | **Section/line number, or reason for not reporting** |
| --- | --- | --- |
| **Study design** | For each experiment, provide brief details of study design including:  a. The groups being compared, including control groups. If no control group has been used, the rationale should be stated.  b. The experimental unit (e.g. a single animal, litter, or cage of animals). | a) method section (statistics paragraph; page 12, lines 261-264) and individual graphs for group comparison  b) method section („statistics“ paragraph; page 12, lines 258-260) |
| **Sample size** | a. Specify the exact number of experimental units allocated to each group, and the total number in each experiment. Also indicate the total number of animals used.  b. Explain how the sample size was decided. Provide details of any a priori sample size calculation, if done. | a) method section („animals“ paragraph; page 6, lines 112-113) and figure legends for detailed information  b) method section („statistics“ paragraph; pages 11-12, lines 255-257) |
| **Inclusion and exclusion criteria** | a. Describe any criteria used for including and excluding animals (or experimental units) during the experiment, and data points during the analysis. Specify if these criteria were established a priori. If no criteria were set, state this explicitly.  b. For each experimental group, report any animals, experimental units or data points not included in the analysis and explain why. If there were no exclusions, state so.  c. For each analysis, report the exact value of n in each experimental group | a) and b) method section („peritoneal contamination and infection sepsis model and PLX5622 treatment“ paragraph; pages 6-7, lines 130-141)  c) information are provided in each figure legend |
| **Randomisation** | a. State whether randomisation was used to allocate experimental units to control and treatment groups. If done, provide the method used to generate the randomisation sequence.  b. Describe the strategy used to minimise potential confounders such as the order of treatments and measurements, or animal/cage location. If confounders were not controlled, state this explicitly | a) method section („animals“ paragraph; page 6, lines 113-114)  b) method section („peritoneal contamination and infection sepsis model and PLX5622 treatment“ paragraph; page 7, lines 139-141 and „NOR“ paragraph; page 11, lines 234-235) |
| **Blinding** | Describe who was aware of the group allocation at the different stages of the experiment (during the allocation, the conduct of the experiment, the outcome assessment, and the data analysis) | method section: page 9, lines 193-194; page 9, lines 203-204; page 10, lines 222-223; page 11, lines 234-235); page 11, lines 254-255 |
| **Outcome measures** | a. Clearly define all outcome measures assessed (e.g. cell death, molecular markers, or behavioural changes).  b. For hypothesis-testing studies, specify the primary outcome measure, i.e. the outcome measure that was used to determine the sample size. | a) detailed information are provided in the method section  b) method section („statistics“ paragraph; pages 11-12, lines 255-257) |
| **Statistical methods** | a. Provide details of the statistical methods used for each analysis, including software used.  b. Describe any methods used to assess whether the data met the assumptions of the statistical approach, and what was done if the assumptions were not met. | a) and b) detailed information are provided in the „statistic“ paragraph in the method section |
| **Experimental animals** | a. Provide species-appropriate details of the animals used, including species, strain and substrain, sex, age or developmental stage, and, if relevant, weight.  b. Provide further relevant information on the provenance of animals, health/immune status, genetic modification status, genotype, and any previous procedures. | a) and b) detailed information are provided in the method section („animals“ paragraph; page 6, lines 112-114) |
| **Experimental procedures** | For each experimental group, including controls, describe the procedures in enough detail to allow others to replicate them, including:  a. What was done, how it was done and what was used.  b. When and how often.  c. Where (including detail of any acclimatisation periods).  d. Why (provide rationale for procedures). | a) – d) detailed information are provided in the method section („animals“ and „peritoneal contamination and infection sepsis model and PLX5622 treatment“ paragraph; pages 6-7, lines 118-150) |
| **Results** | For each experiment conducted, including independent replications, report:  a. Summary/descriptive statistics for each experimental group, with a measure of variability where applicable (e.g. mean and SD, or median and range).  b. If applicable, the effect size with a confidence interval. | a) and b) detailed information are provided in the method section („statistics“ paragraph, page 12, lines 257-258) and in each figure legend |

**Table S1: ARRIVE Essential 10 checklist**
